# Supporting informal caregivers of dementia patients in China: action needed

**DOI:** 10.3389/fpsyg.2025.1661022

**Published:** 2025-10-10

**Authors:** Meiyu Li, Haokun Wang, Yijin Wu, Quan Zhang

**Affiliations:** ^1^School of Economics and Management, China University of Petroleum (East China), Qingdao, China; ^2^School of International Affairs and Public Administration, Ocean University of China, Qingdao, China; ^3^School of Translation Studies, Qufu Normal University, Rizhao, China

**Keywords:** informal caregiver, dementia caregiver, patients with dementia, China, support measures

## Introduction: the growing burden of dementia in China

Dementia, a debilitating disorder, not only affects those diagnosed but also imposes significant strain on their families and caregivers. According to the *2024 China Alzheimer Report*, dementia affects more than 16.9 million individuals, including over 10.6 million individuals with Alzheimer's disease (Wang et al., 2024). By 2050, the number of dementia patients in China is projected to reach 48.98 million, posing a formidable challenge to the healthcare system (Li et al., 2021).

Dementia is characterized by chronic and progressive cognitive decline, leading to severe impairment and high dependency (Reuben et al., 2024). Globally, most dementia patients reside in their homes, with informal caregivers—predominantly family members—providing the majority of care (World Health Organization, 2025). This pattern is also prevalent in China. The overwhelming number of dementia patients surpasses the capacity of nursing homes and long-term care institutions, resulting in the vast majority of patients being cared for at home. Additionally, traditional Chinese cultural values, which place a strong emphasis on filial responsibility, further reinforce this trend, as family members often feel obligated to care for their loved ones with dementia (Wang et al., 2023; Lv et al., 2025). Consequently, 80% of dementia patients in China receive care at home from family caregivers (Li and Wang, 2022).

## The challenges faced by informal caregivers in China

Informal caregivers play a vital role in supporting dementia patients in China; however, this responsibility often places immense burdens on them, negatively impacting their quality of life. The burdens shouldered by caregivers are both multifaceted and profound, encompassing not only the daily challenges of managing complex symptoms and unpredictable—sometimes aggressive—behaviors (Wang et al., 2022; Huang et al., 2025), but also the debilitating financial strain caused by the exorbitant costs of care (Cheng et al., 2025; Lv et al., 2025). Beyond these practical challenges, caregivers often endure significant physical exhaustion and sleep deprivation from the unrelenting demands of caregiving, which can lead to chronic fatigue and deteriorating health (Liu et al., 2022; Mao et al., 2025). Perhaps most insidiously, the psychological toll is the most debilitating—marked by constant emotional strain, feelings of depression, anxiety, helplessness, sadness, and guilt, all of which profoundly affect the mental wellbeing of caregivers (Wang et al., 2022; Huang et al., 2025; Smriti et al., 2024). Notably, negative affective empathy has been shown to intensify this psychological burden (Mei et al., 2025). The cumulative weight of these burdens can be overwhelming, eroding caregivers' quality of life and fostering a sense of isolation, helplessness, and despair (Liao et al., 2024). These challenges have far-reaching effects on the caregivers' own health and wellbeing.

## Lack of support for informal caregivers in China

Despite their critical role, informal caregivers of dementia patients in China receive minimal support (Liu et al., 2024). Although many experience profound psychological and emotional distress, only a small proportion have access to psychosocial services offered by friends, communities, or non-profit organizations (Huang et al., 2025; Cheng et al., 2025). Moreover, caregivers frequently lack essential caregiving knowledge and skills, underscoring the urgent need for structured training programs that can equip them to navigate the multifaceted demands of dementia care more effectively (Wen et al., 2023; Lv et al., 2025). Furthermore, the constant supervision and care required for moderate and severe dementia patients forces many caregivers to abandon their jobs to provide full-time care (Mei et al., 2025; Zhang et al., 2025). Many child caregivers, often referred to as the “sandwich generation” in China, grapple with substantial financial challenges associated with rearing offspring and providing for ailing parents. Yet, dementia patients are largely excluded from China's long-term care insurance policy (Ye et al., 2024), and caregivers seldom receive external financial relief through public programs or external assistance (Guo et al., 2023; Cheng et al., 2025). Moreover, the long-term implementation of the one-child policy has resulted in many family caregivers, particularly children and spouses, shouldering caregiving responsibilities alone, without respite or opportunities for rotation. Yet, few of them receive external support to facilitate rest and recovery, exacerbating their challenges and potentially leading to burnout and a decrease in the quality of care provided to dementia patients (Wang et al., 2019, 2022).

## Current government efforts and their limitations

The Chinese government has initiated some efforts to support informal caregivers of dementia patients, the most notable being the pilot community respite services in cities such as Beijing, Tianjin, and Shanghai (Xinhua, 2025). The municipal government allocates funds to employ formal caregivers from nursing institutions, providing respite services to family caregivers of moderate or severe dementia patients. In 2024, the National Health Commission of China, alongside 15 other government departments, issued the “*National Action Plan for Dementia in Older Adults (2024–2030)*.” This plan outlines strategies to enhance social support for family caregivers of dementia patients, including psychological counseling and respite services, aiming to alleviate caregivers' mental and caregiving burdens (The Central Government of PR China, 2024). However, these initiatives are still in their infancy, and only a limited number of caregivers have access to these services. Current policies in China largely focus on providing support for dementia patients, with insufficient attention to the needs of their caregivers. More comprehensive efforts are required.

## Policy recommendations to support informal caregivers

To enhance support for family caregivers of dementia patients, both the Chinese government and society must take several key actions. First, the government, in collaboration with community centers and social organizations, should increase funding and resources for caregiver support programs, especially mental health services. Innovative interventions such as empathy training, role-playing, mindfulness-based practices, and cognitive behavioral therapy should be explored alongside emerging digital solutions, including online psychological counseling platforms and AI chatbots (Mei et al., 2025; Ruggiano et al., 2021). Second, systematic expansion of knowledge- and skills-based training is critical. Drawing on new technologies and international best practices, the adoption of the WHO *iSupport for Dementia program* represents a feasible and effective strategy for China (Zhi et al., 2024; Brijnath and Antoniades, 2025). Third, policies should be taken to alleviate caregivers' financial burdens. Incorporating dementia-related caregiving services into the reimbursement catalog of long-term care insurance would provide crucial relief. In addition, the government could implement policies such as tax credits or caregiving allowances to help reduce the financial strain. Philanthropic innovations—such as targeted crowdfunding for families facing severe economic hardship—should also be encouraged. Finally, expanding the availability of respite care services and promoting their benefits to a broader group of caregivers would significantly lessen the physical and emotional stress they face (Chen et al., 2022; Kim et al., 2024). Equally important is the effective introduction and dissemination of assistive technologies, which offer significant potential for reducing the physical workload of caregiving (Levenson et al., 2024; Zuschnegg et al., 2025). Notably, recognizing the multidimensional typology of dementia care networks underscores the need for tailored support systems that are specifically designed to address the distinctive and complex challenges faced by informal dementia caregivers (Zhou and Kang, 2025). By supporting informal caregivers, we can not only improve the quality of life for both dementia patients and their caregivers, but also reduce overall healthcare costs and strengthen the national healthcare system. Now is the time to take action and address this pressing issue.

This manuscript presents a holistic synthesis of the multifaceted challenges faced in informal dementia caregiving, situates caregivers' unmet needs within China's evolving policy framework, and advances beyond descriptive analyses by offering actionable, policy-oriented recommendations. It innovatively integrates emerging interventions—such as digital and assistive technologies—with established psychosocial strategies, while positioning the discussion within the transformative context of *China's National Action Plan for Dementia (2024–2030)*. Taken together, these contributions yield a timely, multidimensional, and forward-looking framework for strengthening support for informal caregivers. Looking ahead, future research should engage in cross-national policy comparisons, rigorously assess the applicability and efficacy of innovative supports such as AI-driven tools and online counseling within the Chinese context, and interrogate the heterogeneous challenges confronting caregivers across gender, socioeconomic, and familial dimensions to guide the development of more precisely targeted and sustainable policy responses.
